# Occupational Hazards among Healthcare Workers in Africa: A Systematic Review

**DOI:** 10.5334/aogh.2434

**Published:** 2019-06-06

**Authors:** Sarah Mossburg, Angela Agore, Manka Nkimbeng, Yvonne Commodore-Mensah

**Affiliations:** 1 American Institutes for Research, US; 2 Healthcare Homes, UK; 3 Johns Hopkins University, US

## Abstract

**Background:**

While all healthcare workers are exposed to occupational hazards, workers in sub-Saharan Africa have higher rates of occupational exposure to infectious diseases than workers in developed countries. Identifying prevalence and context of exposure to blood and bloodborne pathogens may help guide policies for prevention.

**Objective:**

This systematic review examined occupational exposure rates to blood and bloodborne pathogen among healthcare workers in sub-Saharan Africa.

**Methods:**

In November 2017, a comprehensive literature search was conducted to identify studies reporting exposure of health workers in African coutnries to blood and bodily fluids. Title, abstract and full text screening were used to narrow our search. Studies more than 10 years old, or published in non-English languages were excluded.

**Findings:**

Fifteen studies reported a variety of exposures. The lifetime prevalence of needlestick injury ranged from 22–95%, and one-year prevalence ranged from 39–91%. Studies included a range of descriptive statistics of knowledge, attitudes, practice and access factors related to exposures. Two studies reported 21–32% of respondents linked poor knowledge or training with prevention of needlestick injuries. Rates of recapping needles ranged from 12–57% in four studies. Attitudes were generally positive toward occupational safety procedures while access was poor.

**Conclusions:**

The high burden of blood and bloodborne pathogen exposures demonstrated here indicates a high risk for contracting bloodborne illnesses. Although the data are sparse, implementation of preventative policies based on current knowledge remains critical to minimize risk and reduce exposure. There remains a pressing need for high quality data on occupational hazards to identify the burden of exposures and inform preventive policies in Sub-Saharan Africa. Additional studies are needed to determine whether differential exposures exist between professions and the associations with knowledge, attitudes, practices, and access factors to create targeted strategies to diminish occupational hazards.

## Introduction

Healthcare workers provide patient care in environments that are considered to be one of the most unsafe occupational settings [1,2]. Occupational hazards that include biological, chemical, physical, ergonomic, psychosocial, fire and explosion, and electrical hazards [3] threaten healthcare worker lives, safety, and well-being. Globally, it is estimated that 1 in 10 healthcare workers, experience a sharp injury every year [4]. In the year 2000, sharps injuries to healthcare workers resulted in 16,000 hepatitis C virus (HCV) infections, 66,000 hepatitis B virus (HBV) infections, and 1,000 human immunodeficiency virus (HIV) infections. The impact of these infections is significant. Between 2000 and 2030, these infections are estimated to cause 145 premature deaths due to HCV, 261 premature deaths due to HBV, and 736 premature deaths due to HIV [5]. In sub-Saharan Africa, the limited studies conducted have demonstrated that healthcare workers are frequently exposed to biological, chemical, and physical occupational hazards [6,7].

HBV, HCV and HIV prevalence among healthcare workers who experience sharp injuries highlight the disproportionate burden that sub-Saharan Africa bears in contrast to developed countries. For instance, in the Africa E sub-region (including Botswana, Congo, Malawi, South Africa etc.),11.8% of HBV, 2.8 of HCV, and 5.1% of HIV infections are attributable to occupational exposure [5]. This is in sharp contrast with the America A sub-region (Canada, Cuba, United States) where 0.51% of HBV, 1.6% of HCV and 0.29% of HIV infections are attributable to occupational exposure [5]. The higher prevalence is partly explained by the higher prevalence of bloodborne pathogens in the general population but can also be attributed to poor healthcare infrastructure in sub-Saharan Africa [8,9].

There are well-established guidelines to prevent exposure to occupational hazards, including blood and bloodborne pathogens. These include educating healthcare workers on safer use of devices, procedures and management of exposures. Furthermore, the World Health Organization (WHO) has instructed governments to transition to the exclusive use of safety injection devices by 2020 [10]. While developed countries have heeded this recommendation, the vast majority of sub-Saharan African countries have failed to enact legislation to protect healthcare workers. Apart from provider behaviors that increase exposure to occupational hazards, system-level barriers increase the risk of exposure to hazards in the healthcare setting. Unsafe conditions in the healthcare environment, lack of personal protective equipment (PPE), and high provider to patient ratio increase the risk of exposure to bloodborne pathogens and cause preventable infections. Healthcare workers in four African countries (Cameroon, South Africa, Uganda and Zimbabwe), have reported that the top four reasons for migrating to developed countries include better remuneration, safer work environment, living conditions and lack of facilities [11]. The 2006 World Health Report Working Together for Health drew attention to the severe healthcare worker shortages in 57 countries, most of them in Africa and Asia [12]. The influence of occupational hazards on healthcare worker shortages in sub-Saharan Africa has dire implications for patient outcomes, productivity and life expectancy in the continent.

Better understanding of the occurrence of occupational hazards among healthcare workers in sub-Saharan Africa can inform policies to make the healthcare environment safer for healthcare workers. Hence, the purpose of this systematic review was to examine the occurrence of exposure to blood and bloodborne pathogens among healthcare workers in sub-Saharan Africa.

## Methods

### Search Strategy

The primary outcome for this systematic review was healthcare worker exposure rates to bloodborne pathogens. Secondary outcomes included knowledge, attitudes, practices, and access factors that potentially contributed to exposures. We conducted a broad literature search using occupational health terms that were most likely to capture studies in this area, and then narrowed our search via title, abstract and full text screening. The literature search was conducted in November 2017. Three databases (PubMed, Embase, and Cumulative Index to Nursing and Allied Health Literature [CINAHL]) were searched for (“safety” or “chemical safety” or “equipment safety” or “fire safety” or “occupational safety” or “radiation safety” or “occupation*” within three words of “safety or hazard*” or “hazard*” or “accident*”) AND (“Health personnel+” or “nurse*” or “physician*” or “doctor*” or “surgeon*”) AND (“Africa South of the Sahara” or “Africa South of the Sahara” or “central Africa”) and all sub-Saharan African countries.

**Figure 1:**
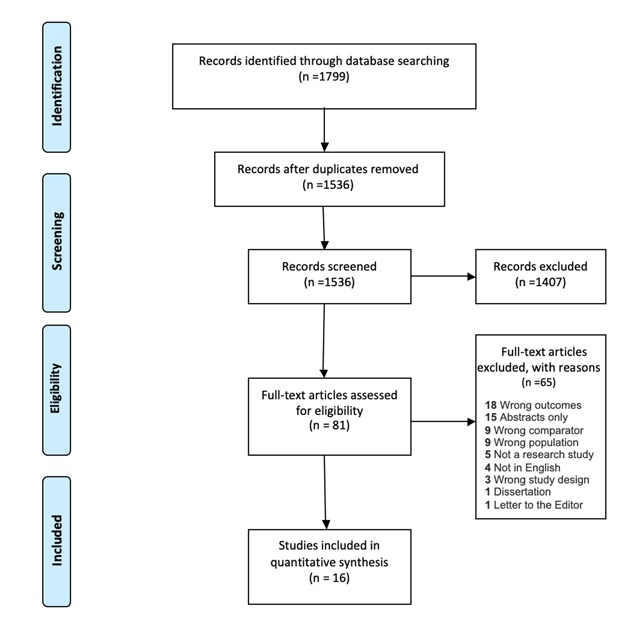
Prisma diagram for search strategy.

A total of 1799 references were retrieved from searches (Figure 1). There were 263 duplicates removed and 1536 articles were left for screening. Of these, 1407 studies were excluded during abstract screening. Studies were included if they were in English, involved occupational exposure to blood and bodily fluid, and included healthcare workers in an African Country. Studies were excluded if they were published more than 10 years ago. Eighty-one articles were forwarded to full text screening and 65 of those were excluded. Figure 1 includes reasons for full text exclusion. Screening was conducted by two independent reviewers and conflicts were resolved through consensus at each stage.

### Data extraction

Fifteen articles met the inclusion criteria and were extracted for synthesis. Three reviewers (AB, SM &MN) conducted the extraction for this review. Each article was extracted by two independent data extractors. Data was extracted using a pre-determined table created to extract data relevant to this review. Disagreements in extracted content were resolved through detailed review of the article, discussion and consensus by all team members (AB, SM, MN & YCM).

## Results

Descriptive data for each of the reviewed studies including country, study population, sample size, sampling strategy, response rate and type of occupational exposure are presented in Table 1. Studies took place in a single country with the exception of one study involving surgeons from 14 African countries [13]. Nigeria had the most published studies (n = 4), followed by South Africa (n = 3), Ethiopia (n = 2), and Uganda (n = 2). One study each was conducted in Cameroon, Kenya, Sudan, and Tanzania. There were a range of disciplines represented, one study included only nurses [14], five studies included only physicians [15,16,17,18,19], and the remaining ten studies included multiple disciplines [20,21,22,23,24,25,26,27,28,29]. Sample sizes ranged from 30–843, and response rates ranged from 41–94%, although four studies did not report response rates.

**Table 1:** Descriptive data of included studies.

Author, Year	Country	Design	Population	Sample Size	Sampling Strategy	Response Rate (%)	Type of Occupational Health Exposure	Limitations (Per Authors)

Mathewos et al., 2013	Ethiopia	Cross-sectional	Doctor, nurses, laboratory technician, health officer, Anesthetics, Midwives and Physiotherapists	195	Random	NR	Bloodborne pathogen and body fluid	NR
Aminde et al., 2015	Cameroon	Cross-sectional	Nurses	80	Convenience	94	Bloodborne pathogen	Cross-sectional design, small sample size
Ogoina et al., 2014	Nigeria	Cross-sectional predictive correlational	Nurses, physicians and laboratory scientist	290	Convenience	76	Bloodborne pathogen, Body fluid	Measurement Error, Recall Bias
Manyele et al., 2008	Tanzania	Cross-sectional	Nurses, Physicians, medical attendants	430	Randomly selected	NR	Bloodborne pathogen, body fluid	NR
Ndejjo et al., 2015	Uganda	Cross-sectional descriptive	Nurses, Physicians, Midwives, clinical officers	200	Random	NR	Bloodborne pathogen	Recall Bias, cross sectional study, one facility which limits generalizability
Kumakech et al., 2011	Uganda	Cross-sectional descriptive	Nurses, Physicians, Midwives, Medical lab techs and students (nursing and medical)	224	Stratified systematic sampling	58.3	Bloodborne pathogen, Body fluid	Measurement Error, Recall Bias, Involvement of students
Aluko et al., 2016	Nigeria	Cross-sectional	Nurses, Physicians, Nursing Assistants	290	Stratified sampling and simple random sampling	93	General	Cross sectional design, response bias, lack of generalizability
Engelbrecht et al., 2015	South Africa	Cross-sectional descriptive	Nurses, Physicians, Nursing Assistants, Allied health professionals (Social workers, physiotherapists, radiographers and dieticians)	513	Purposive, stratified quota	46	Bloodborne pathogen, Body fluid	Selection Bias, non-probability sampling
Efetie et al., 2009	Nigeria	Cross-sectional survey	Physicians	72	Convenience	72	Bloodborne pathogen	Selection Bias, small sample size
Phillips et al., 2007	Cameroon, Ethiopia, Ghana, Kenya, Madagascar, Malawi, Mozambique, Nigeria, Rwanda, South Africa, Sudan Tanzania, Uganda, Zambia	Cross-sectional	Physicians (Surgeons)	84	Convenience	76	Bloodborne pathogen, Body fluid	NR
Bekele et al., 2014	Ethiopia	Cross-sectional descriptive	Physicians (Surgeons)	98	Convenience	75	Bloodborne pathogens	Measurement Error, Recall Bias, Too small sample size
Nwankwo et al., 2011	Nigeria	Cross-sectional	Physicians (trainee surgeons)	184	Convenience	80	Bloodborne pathogens	NR
De Silva et al., 2009	South Africa	Cohort	Physicians, Surgical Assistants	30	Random	41	Bloodborne pathogen, body fluid	Small sample size
Karani et al., 2011	South Africa	Cross-sectional	Physicians(Interns)	53	Convenience	83	Bloodborne pathogen, Body fluid	Too small sample size, Recall bias of participants. Limited to MDs only.
Ogendo et al., 2008	Kenya	Cross-sectional	Surgeons and first assistants	346	Convenience	NR	Bloodborne pathogen	Selection Bias, Measurement Error

*Note:* NR = Not reported.

### Prevalence

Our primary outcome of interest was prevalence or incidence of blood and bloodborne pathogen exposure from needlestick injuries or muco-cutaneous exposures. Studies reported a variety of prevalence or incidence rates (Table 2). Current lifetime needlestick injury prevalence ranged from 22–95% [15,16,24,26,29]. One study reported two-year incidence rate of needlestick injuries of 21% [22]. Four studies reported a one-year incidence rate ranging from 39–91% [13,17,23,28]. Two studies reported overall rates of accidental blood exposure via multiple methods (needlestick injuries, non-sharps, splashes, etc.). Of these, one reported a six-month overall accidental blood exposure incidence rate of 68% [18], while the other reported a one-year overall accidental blood exposure incidence rate of 84% [28]. Two studies were more narrowly focused on HIV exposure, reporting prevalence rates of exposure to HIV (68%) [14] and HIV risky conditions (34%) [25]. Three studies reported one year incidence of muco-cutaneous exposures ranging from 24–76% [17,23,28]. Two studies designed to evaluate splash rates on protective eyewear in the operating room during procedures reported 45–53% of eyewear was splashed [20,27].

**Table 2:** Prevalence of needlestick injuries and muco-cutaneous exposures to blood and bloodborne pathogens experienced by healthcare workers in Sub-Saharan Africa.

Author, Year	Primary Outcome	Primary outcome prevalence (%)	Independent Variable	Factors related to knowledge	Factors related to attitudes	Factors related to practices	Factors related to access

Aluko et al., 2016	Knowledge, attitudes and practices on occupational exposures, risk and history of injury and prophylaxis	Perceived susceptibility to needle stick injuries 94.5%, body fluid contact 92.4%	None	57.6% had high knowledge of occupational hazards, 42.6% low knowledge of occupational hazards, 58% acquired through professional training, 67% aware of job aids, 93% aware of PEP	80% had positive attitudes towards occupational hazards and preventive safety practices; Reasons for non-compliance with safety equipment: 6% report waste of time, 1% report uncomfortable as	96% report wearing gloves for routine clinical practice, 94% reporting safe sharps disposal, 52% always comply with standard safety precautions	41% report lack of safety equipment as a reason for non-compliance with safety equipment
Aminde et al., 2015	Knowledge of PEP for HIV	68% lifetime HIV occupational exposure: 24% both needlestick and splash exposure, 63% needlestick only; 1-year incidence: 54% had 1 exposure, 32% had 2 to 3, 15% >4 exposures	Demographics, length of service, previous formal training, hospital policies and source of knowledge	84% had heard about PEP, 99% correctly identified the appropriate first aid measure, 30% correctly stated expanded 3 drug regimen for PEP and only 25% knew correct duration for therapy; Reasons for no PEP: 9% unaware of need, 16% unaware of hospital PEP policy	86% perceived they were at risk HIV acquisition, 18% did not receive PEP because believed no susceptibility to HIV	Recapping needles 37%	2% PEP no available
Bekele et al., 2014	Hepatitis B vaccination	78% prevalence of needle stick injury, 23% received HBV vaccine	Demographics	19% report not vaccinating due to not knowing vaccine available in Ethiopia	94% believed Surgeons should get HBV vaccination, 49% report reason for not vaccination was “I didn’t give it much thought in the past”, 14% report not vaccinating because it was time consuming, 8% report not vaccinating because they believed it was not useful as a Surgeon	24% HBV vaccination rate, of those 75% (18/24) received all doses; 39% double gloved during procedures, 57% inconsistent double gloving, 4% never double gloved	14% report not vaccinating due to cost
De Silva et al., 2009	Risk of blood splashes to the eyes during surgery	45% of visors had blood splashes, of these 68% (15/22) had macroscopic splashes, 73% (16/22) had microscopic splashes	Major/minor surgery, emergency/elective surgery, surgeon/assistant, use of special equipment	NR	NR	No significant differences identified	NR
Efetie et al., 2009	Prevalence of needlestick injuries	90% lifetime needle stick injuries	Type of hospital, Physician rank	NR	NR	16% from recapping; 51% recapped needles by hand, 56% indicating regular use of sharps containers; 9% took appropriate action after needlestick injury, 52% didn’t report needlestick injury, 9% (6/68) took ARV; 92% indicate double-gloving	69% indicated presence of sharps disposal containers, 37% reported needlestick policy at work
Engelbrecht et al., 2015	Health and safety practices, prevention of blood and air-borne diseases	21% needlestick injury or exposure to body fluids (2 years)	Demographics, occupation, trust in management	Lack of training reported: 24% on use of PPE, 21% prevention of needlestick injuries	NR	57% recap needles, 29% washed gloves, 20% didn’t wash hands between patients	Infection control hazards present in all three hospitals observed (i.e. no soap, sharps containers overflowing, N95 masks not available, etc.)
Karani et al., 2011	Accidental exposure to blood or body fluids	55% exposure to blood or body fluids (1 year), 72% (21/29) were percutaneous exposures, 24% (8/29) were mucosal exposures	None	NR	NR	88% (23/26) compliance with PEP prophylaxis when HIV positive exposure. PEP discontinued due to intolerance of medication side effects	NR
Kumakech et al., 2011	Occupational exposure to HIV (percutaneous injury and muco-cutaneous contamination)	39% needlestick injury (1 year), 3% scalpel cut injuries (1 year), 58% muco-cutaneous exposure (1 year)	Demographics, predisposing factors to exposure	32% poor clinical knowledge contributed to NSI	NR	12% recapping needles; 10% being less careful; 2% improper sharps disposal; 47% reported exposure; 5% PEP initiated and completed	NR
Manyele et al., 2008	Availability of information on occupational health and safety (OHS), availability of qualified OHS supervisors, quantify hazardous activities in the hospital, distribution of accidents in hospitals	Needle stick injuries 52.9%, blood splashes 21.7% (timeframe not reported)	None	33% report seminars and workshops as highest source of information about OHS	NR	Hazardous activities identified included injection, cleaning, patient care, bedding, dressing of wounds, medication and operation.	Hospitals in Kagera, Lindi, and Mawenzi had accessibility of antiseptics to less than 30% of health service providers.
Mathewos et al., 2013	Knowledge level of the HCWs about PEP for HIV	33.8% exposed to HIV risky conditions (lifetime)	None	63.1% had adequate knowledge about PEP for HIV, 48.7% received this in formal training, 60.5% reported that PEP is efficient and 50.7% knew when to initiate PEP	98.5% agreed on the importance of PEP for HIV, 78.5% believed it can reduce probability of being infected	Of the exposed, 74.2% (49/66) took PEP; of those who took PEP, 79.5% (39/449) completed PEP	88.2% reported availability of PEP guidelines in the hospital.
Ndejjo et al., 2015	Biological and non-biological occupational hazards	21.5% sharp-related injuries, 17% cuts and wounds, 10.5% direct contact with contaminated specimens/biohazards, 9% airborne diseases, 7.5% infectious diseases, 7.5% other bloodborne pathogen, vector-borne disease, and bioterrorism (time not reported)	Demographics, provider specialty, overtime work, type of facility, alcohol consumption and sleep	NR	97.0% were screened for HIV	Biological hazards associated with not wearing necessary PPE (AOR = 2.34, p = 0.006), working overtime (AOR = 2.65, p = 0.007), and experiencing work related pressure (AOR = 8.54, p = 0.001); 79.5% washed their hands before and after every procedure; 68.5% washed after handling soiled materials; 46% washed when evidently dirty; 53.5% washed after using the toilet; 44.3% (35/79) of those exposed wore all necessary PPE	Availability of medical waste disposal (92.0%); safety tools and equipment (90.0%); PPE provided by hospital (53.5%)
Nwankwo et al., 2011	Percutaneous injuries and accidental exposure to patient’s blood; knowledge of universal precautions and post-exposure prophylaxis	68% accidental blood exposure (6 months); of those 64% (89/140) needlestick injuries, 24% (33/140) blood splashes and non-sharp, 10% (14/140) operating instrument injuries, 3% (4/140) from surgical blades	Demographics, surgical specialty, Physician rank	42% adequate knowledge of universal precautions and PEP	NR	Post-exposure practices: 54% wash with water and clean with spirit, 6% cleaned with hypochlorite solution, 72% disregarded exposure, 1% took ART	NR
Ogendo et al., 2008	Blood splashes on eyewear	53.1% contamination rate protective eyewear, 5.2% of surgeons and 3.5% assistants utilized eye protection	Demographics, use of power tools	NR	Reasons for not wearing goggles: 33% uncomfortable, 26% unavailable, 17% misting, 2% using headlamp or prescription glasses, 2% forgot or unaware	Longer surgeries and use of power tools had more splashes	NR
Ogoina et al., 2014	Needle stick injuries, cut by sharps, blood splashes and skin contact with blood	84.4% had > = 1 exposure (1 year): 44.7% needlestick injury, 32.8% cuts by sharps, 33.9% blood splashes, and 75.8% skin contact with blood	Demographics	48.6% had training in infection control	NR	NR	NR
Phillips et al., 2007	Bloodborne pathogen exposure, body fluid exposure, access and use of protective equipment	91% percutaneous injury in the last year, mean 3.1 exposures80% > = 1 blood and body fluid exposure in the last year, mean 4.2 exposures	None	NR	NR	39% vaccinated against HBV; 40% used hands-free technique for passing sharps; 31% used blunt suture needles; 82% typically wear a gown during surgery, 35% reported wearing a gown during most recent exposure; 29% report wearing eye protection.	89% had access to PEP

*Note:* NR = Not reported, NA = Not applicable, AOR = Adjusted odds ratio.

Secondary outcomes were factors related to knowledge, attitudes, practices and access that predispose healthcare workers to blood and bloodborne pathogen exposures (Table 2). Most studies reported descriptive data (percentages) rather than inferential tests of associations of these factors with exposures. We found that practice factors were the most commonly included (13 of 15 studies) followed by knowledge and access factors (each with 8 of 15 studies) and finally attitude factors (6 of 15 studies).

### Knowledge

General knowledge or training was described in two studies where 21–32% of respondents reported either a lack of training or poor knowledge related to prevention of needlestick injuries [22,23]. Several studies explored knowledge factors as they related to post-exposure prophylaxis (PEP) [22,23]. Four studies reported between 42–93% of those surveyed had adequate knowledge of PEP [14,18,25,29]. While one study reported that 24% of those surveyed lacked adequate training on use of PEP [22]. Respondents with some type of formal training on PEP, HIV exposure or occupational health exposures ranged from 33–49% [24,25,28].

### Attitudes

Of the eight studies describing attitude factors, three indicated that 80–99% of participants reported positive attitudes towards occupational safety measures [16,25,29]. In two of these studies, there were a small minority (6–8%) of healthcare workers surveyed that reported not following safety procedures because they didn’t perceive them to be useful [16,29]. Reported reasons for noncompliance with safety practices often fell into the category of attitude factors. In two studies, 1–33% of respondents indicated discomfort was a reason for non-compliance with safety equipment [27,29]. Two studies also reported that 6–14% of respondents indicated time was a factor for noncompliance [16,29].

### Practices

A number of practices related to exposure of healthcare workers to blood and bloodborne pathogens were reported across studies. Rates for recapping needles ranged from 12–57% in four studies [14,15,22,23]. Two studies reported that 94–98% of study respondents properly disposed of sharps [29]. The practice of taking PEP after an exposure varied widely, ranging from 1–88% in five studies [15,17,18,23,25]. The two studies that included information about participant reporting rates after exposure were consistent with 47–48% reporting exposures [17,23].

### Access

Some of the studies also reported on factors that could be grouped into access to safety equipment. Between 2–70% of participants in four studies indicated that some type of safety equipment or PEP was not available to them [14,24,26,29]. Two studies assessed the availability of occupational health policies, with 37–88% reporting polices were available [16,25]. Finally, two studies indicated that 69–92% of participants had sharps or waste disposal available to them at their hospital [15,26].

## Discussion

This review sought to identify studies that examined occupational exposures to blood and body fluids in healthcare workers and potential factors predisposing workers to exposures in sub-Saharan Africa. Our review identified a high burden of occupational hazards as well as knowledge, attitude, practice and access factors among healthcare workers representing different professions.

Several of the prevalence estimates of occupational hazards had a wide range. Lifetime prevalence of needlestick injuries spanned from 22–95% in the five studies reporting this statistic. These differences may be partially explained by variations in sampling among the studies. Isolating the studies that included only physicians (surgeons and gynecologists) shows that they both had high rates, although relatively small sample sizes (n < 100) [15,16]. Surgeons have high exposure rates to sharps in the operating room, which may increase the likelihood of a needlestick or other sharp injury. Similarly, if gynecologists are also practicing as obstetricians and performing cesarean sections, or performing gynecological surgeries in the operating room they may also have higher exposures to sharps. Of the remaining studies that included a diverse sample of healthcare workers, the study with the highest lifetime prevalence rate (95%) was in a single hospital in Nigeria [29]. It is possible that this hospital is an outlier with a high rate for a variety of contextual reasons that are not immediately apparent. The two studies with the lowest prevalence rates (<53%) each took place in multiple hospitals with a more diverse sample of healthcare workers including physicians, nurses, nursing assistants, midwives, and clinical officers [24,26].

The one-year incidence of needlestick injuries ranged from 39–91%. These differences may also be related to the population that was sampled in the respective studies, although this is unlikely to account for all the variation in rates. Similar to the high lifetime prevalence reported in surgeons above, the study that included only surgeons had the highest incidence [19]. Again, this may be partially explained by the high exposure rates surgeons have to sharps in the operating room. The study that reported the lowest one-year incidence included students, who may have a lower incidence rate because as a student they have lower exposures in general [23].

While the prevalence and incidence findings have limited applicability because of their significant variability, the qualitative findings concerning knowledge, attitudes, practices, and access factors provide critical information to help inform prevention strategies. While the amount of data in the studies we examined concerning these factors varied widely, it begins to present a picture of potential provider and system issues that may be contributing to sub-Saharan African healthcare workers’ exposures to blood and body fluid. Provider knowledge does appear to be a contributing factor to some extent. It is troubling that 21–32% of respondents linked the lack of training and poor knowledge to prevention of needlestick injuries in two studies [22,23]. There was also a notably wide range of adequate knowledge in PEP practices (42–93%). So even if PEP is available, it is likely that providers with inadequate knowledge may not complete PEP treatment.

While there were generally positive attitudes towards preventative safety practices reported, albeit in only three studies, there were some negative attitudes worth noting. A small percentage of respondents (6–8%) were non-compliant with safety equipment or practices because they did not consider them useful. More in-depth exploration of these beliefs could provide data for potential interventions. One study reported a high rate of discomfort as a reason for not using goggles in the operating room. Because this finding was only observed in one study, and a single piece of equipment, it is unclear whether discomfort is a common reason for non-compliance with other safety equipment use. This finding is worth exploring in future research.

Considering the wide range in knowledge about PEP, it is not surprising that there was a range in practices in taking PEP (1–88%), although only two studies collected data about knowledge of PEP and practice in taking PEP. The results from these studies appear to be discordant. One study reported 42% adequate knowledge, yet only 1% PEP [18], while the other study reports 63% adequate knowledge and 74% PEP [25]. It is difficult to draw meaningful conclusions from such sparse data. Among the five studies that reported PEP uptake, there was no clear contextual factor that explained the wide range. Three out of five of the studies took place in an individual hospital, it is possible that contextual factors within each hospital account for the higher or lower rates of PEP uptake.

Preventing and mitigating occupational hazards among healthcare workers in sub-Saharan Africa requires a systematic approach to providing occupational safety and health at the national, district and facility levels with careful integration into outbreak preparedness plans. The protection of healthcare workers requires institutionalization of occupational health risk assessment and risk-based medical surveillance. In the recent inter-country workshop on occupational safety and health in Africa [30], it was identified that many countries lack national regulations for occupational safety and health which cover public health care facilities. A call was made for all sub-Saharan African countries to develop regulations, standards, and management according to the WHO/International Labor Organization global framework. This strategy will likely have the most significant and sustained impact on managing occupational safety and health in sub-Saharan Africa [30].

Low levels of knowledge demonstrated by healthcare workers in this review call for policies that create a culture of awareness of occupational hazards and their influence on patient outcomes. These policies may include mandatory workshops and training on occupational hazards and dedicated occupational health units at healthcare institutions. These units may address the inadequacies in the safe provision of health services, occupational hazards, and statistics on the healthcare environment to ensure that healthcare workers are adequately rehabilitated and protected.

There is a dire need for national policies to address insufficient and in some cases absence of PPE in many sub-Saharan Africa countries. When worn correctly, PPE provides a barrier to protect healthcare workers from exposure to contaminated body fluids which may contain infectious agents. At the basic level, PPE protects the hands, eyes, nose, and mouth and includes equipment such as boots, gloves, and face shields. Extended PPE includes impermeable gowns, head covers and face masks. In Akagbo et al.’s study among healthcare workers in Ghana, 74% reported that sometimes PPEs were not available but also stated that donning PPE during emergencies would result in adverse outcomes or death or cause patients to panic [31]. This scenario paints a complex picture of why PPE may not be used consistently. Use of PPE may result in significant physiological or physical stresses to healthcare workers. The most common stress associated with PPE in the African context is heat stress which may limit compliance, performance and could be life-threatening [32,33]. The standards for the production of PPEs should therefore be reevaluated to take into consideration the warmer climate in Africa to promote adherence.

To our knowledge, this is the first examination of occupational exposure to bloodborne pathogens in sub-Saharan Africa. The examination of knowledge, attitudes, practices and access factors may inform strategies to reduce exposures in diverse clinical settings. There are some limitations to our review worth noting. It is possible that the data presented here for PEP under-represents published data as PEP was not the primary focus of the review. We did not include a formal method for evaluating the quality of the studies that we included, rather because there were so few studies on the topic chose to include all available published data. Many studies grouped together healthcare workers from disparate professions with varying opportunities for exposure which could affect needlestick injury or muco-cutaneous exposure rates. These sampling strategies limit us from developing a deeper understanding of prevalence within distinct professions. Further research exploring rates within different professions would be helpful to build a basis for targeted interventions in these heterogeneous groups.

## Conclusion

This study identified a high burden of needlestick injuries and muco-cutaneous exposures to blood and bloodborne pathogens for healthcare workers in sub-Saharan Africa. This finding indicates that these healthcare workers are at high risk of contracting bloodborne illnesses such as HCV, HBV, and HIV. This review identified that sparse data exists exploring factors correlated with these exposures and inconsistent research among studies which explored these factors. The development of effective interventions to counteract causes of increased prevalence and incidence of needlestick injuries or muco-cutaneous exposures is necessary even in light of the limited available knowledge of factors influencing these rates.
